# Emerging Governance Principles for Health‐System Transformation in the Digital Era: A Policy Discourse Analysis of an Official Side Event of the Seventy‐Ninth World Health Assembly

**DOI:** 10.1002/hcs2.70110

**Published:** 2026-10-26

**Authors:** Hui Yin, Xiaobaiju Wu, Daping Song, Yunping Wang, Xiaoyun Liu, He Zhu, Xueqing Tian, Xiaodan Fan, Yingpeng Qiu, Minghui Ren

**Affiliations:** ^1^ Department of Global Health, School of Public Health Peking University Beijing China; ^2^ Institute for Global Health Peking University Beijing China; ^3^ China National Health Development Research Center Beijing China; ^4^ China Center for Health Development Studies Peking University Beijing China

**Keywords:** governance principle, health‐system transformation, official side event, policy discourse analysis, World Health Assembly

## Abstract

**Background:**

Health‐system transformation has become a major policy priority in response to demographic change, rising chronic disease burdens, workforce constraints, and fiscal pressures. Although digital technologies are widely promoted as enablers of transformation, less attention has been paid to the governance principles that guide their implementation and integration within health systems.

**Methods:**

This study employed a qualitative policy discourse analysis of an official side event held on May 18, 2026, during the Seventy‐ninth World Health Assembly. The complete transcript of 16 speakers was analyzed using a structured coding framework comprising four dimensions: challenge framing, principle justification, transformation pathways, and envisioned health systems. Actor type and van Leeuwen‐aligned legitimation strategies were also coded. A master governance principles matrix, theme frequency tables, actor‐theme heatmaps, and discursive convergence assessments were generated to synthesize the findings.

**Results:**

Five interrelated, discourse‐grounded analytical constructs emerged: equity‐oriented transformation, people‐centered digital health, governance‐led system architecture, evidence‐based and context‐sensitive implementation, and national ownership with international cooperation. Digital tools were framed as supportive instruments rather than primary drivers. Actor‐specific emphases varied, including less explicit people‐centered language among World Health Organization headquarters speakers and more frequent financing and system‐architecture language in that group. Broad discursive convergence, rather than formal policy consensus, was observed within the event.

**Conclusions:**

Official side events offer a useful but context‐specific lens to examine how governance principles for health‐system transformation are constructed, justified, and operationalized. Findings highlight the role of governance frameworks in enabling effective, equitable, and sustainable transformation, while recognizing that convergence in diplomatic discourse should not be interpreted as formal policy consensus or implementation commitment.

## Graphical abstract

Five governance principles for health‐system transformation emerged from policy discourse at an official Seventy‐ninth World Health Assembly side event. The study demonstrates how shared governance norms are constructed and legitimized through multilateral dialog among governments, World Health Organization representatives, and technical experts.

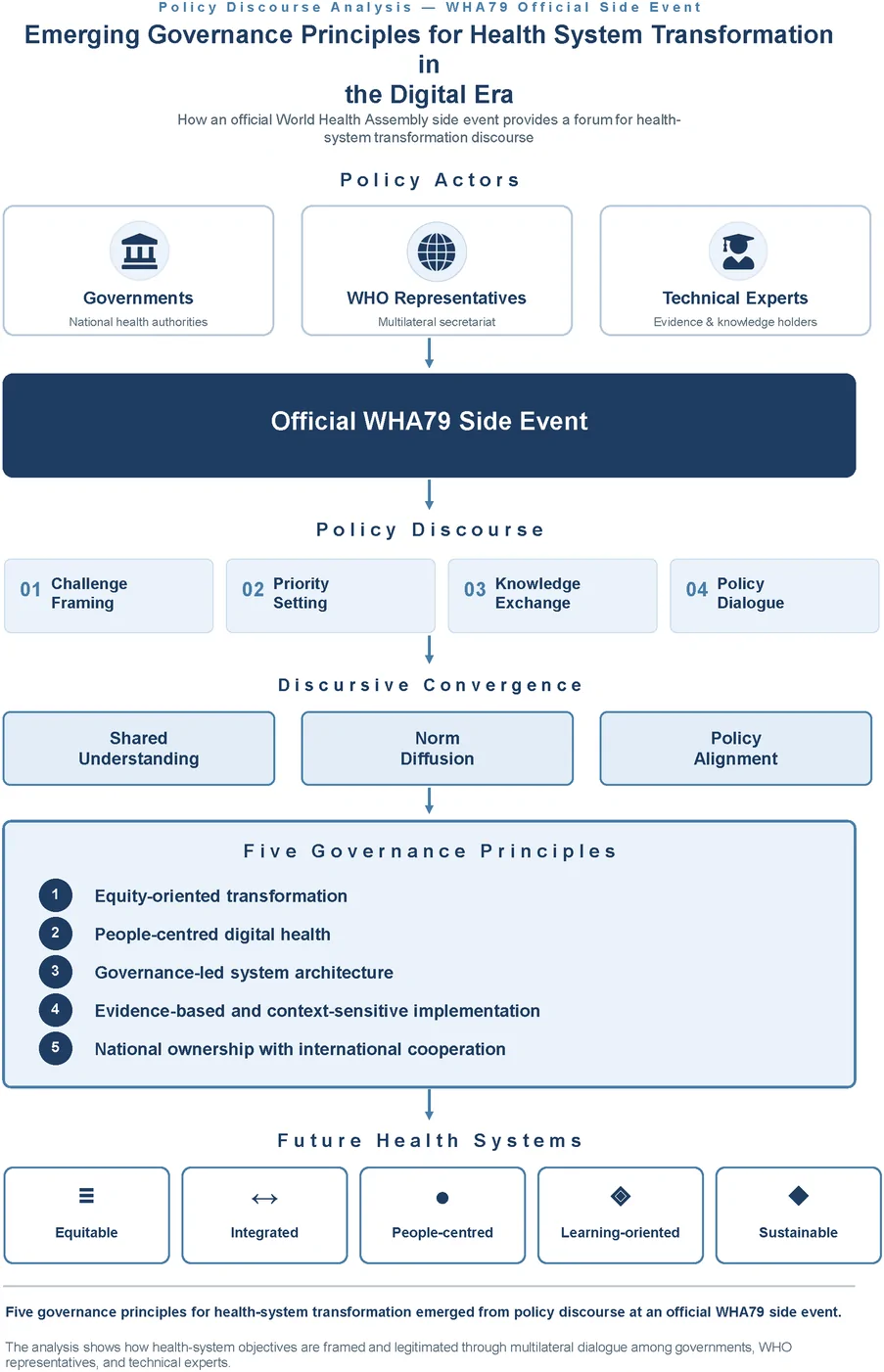

**Abbreviations**
AI: artificial intelligence
DPI: digital public infrastructure
H: high
HQ: headquarter
L: low
LMIC: low‐ and middle‐income country
M: medium
P1: equity‐oriented transformation
P2: people‐centered digital health
P3: governance‐led system architecture
P4: evidence‐based and context‐sensitive implementation
P5: national ownership with international cooperation
PHC: primary health care
WHA: World Health Assembly
WHA79: Seventy‐ninth World Health Assembly
WHO: World Health Organization

## 1 Background

Transforming health systems has become a critical priority for countries worldwide, driven by demographic changes, rising chronic disease prevalence, workforce constraints, and fiscal pressures [1, 2]. In the digital era, emerging technologies offer opportunities for improving service delivery and coordination, but they may also create potential inequities if governance and implementation processes are inadequately designed [3, 4]. While technological innovation receives considerable attention, the governance arrangements necessary for effective, equitable, and sustainable health‐system transformation remain less understood.

Existing research has largely focused on technology adoption, including artificial intelligence (AI), telemedicine, and health data platforms [5, 6], with limited attention to how governance principles—including normative frameworks, institutional arrangements, and alignment‐building mechanisms—shape implementation and operationalization. Understanding how policy actors collectively interpret and legitimize these principles in multilateral settings is essential.

The World Health Organization (WHO) promotes health‐system transformation through normative guidance, capacity‐building programs, and multilateral forums. Official side events at the World Health Assembly (WHA) provide institutionally recognized spaces for policy dialog, allowing participants to articulate challenges, propose priorities, and discuss implementation strategies. These events offer a valuable opportunity to examine how governance principles are constructed, justified, and linked to broader visions of future health systems.

This study addresses two questions. First, what governance principles for health‐system transformation emerge from policy discourse at an official side event of the Seventy‐ninth WHA (WHA79)? Second, how are these principles justified, operationalized, and connected to broader visions of equitable, integrated, and sustainable health systems by different policy actors? To answer these questions, we applied policy discourse analysis to the complete transcript of the official side event and examined relationships among challenge framings, governance principles, implementation pathways, and envisioned system characteristics.

Health‐system transformation extends beyond technological adoption and is fundamentally a governance challenge requiring the alignment of normative objectives, institutional arrangements, and implementation strategies. Governance principles provide the framework through which policy priorities are defined, interventions are justified, and accountability is maintained, ensuring that health‐system reforms achieve equitable, integrated, and sustainable outcomes [7]. In this study, governance principles are treated as analytical constructs grounded in the discourse, not as formal WHO principles or universal normative recommendations.

Policy discourse is a primary mechanism through which governance principles are constructed and communicated [8]. Official side events at the WHA provide institutionally recognized platforms for policy actors to articulate challenges, negotiate priorities, and justify strategies. These discussions reveal how actors collectively interpret governance principles, reconcile differing perspectives, and link high‐level objectives to concrete implementation pathways.

Existing frameworks already articulate many elements relevant to this analysis, including WHO health‐system strengthening, workforce, digital health, and AI‐governance guidance, as well as broader work on governing digital health futures [9, 10, 11, 12, 13, 14]. This study therefore does not claim that the five principles are wholly new. Rather, it examines how established norms were recombined, prioritized, and legitimized in a specific WHA79 side event discourse. Two patterns were less explicit in earlier framework documents but prominent in this dataset: the repeated governance‐before‐technology framing and the coupling of national ownership with digitally mediated international cooperation.

Analyzing discourse in these settings enables the identification of recurring patterns in how governance principles are articulated and operationalized. This approach situates the study within the empirical investigation of governance processes, distinct from purely theoretical research, and provides a foundation for examining the official side event of the WHA79 as an empirical case.

## 2 Methods

### 2.1 Study Design and Data Source

This study employed a qualitative policy discourse analysis to examine how governance principles for health‐system transformation are articulated, justified, and legitimized through policy dialog. The empirical case was an official side event held on May 18, 2026, during WHA79. It was selected as an information‐rich institutional case because it brought together national governments, WHO headquarters, WHO regional offices, and technical experts in a formal multilateral policy setting focused on health‐system transformation in the digital and intelligent era [15]. The analysis does not treat the event as statistically representative of all WHA deliberations; rather, it examines how governance language was produced in this specific institutional setting. The 18 speakers comprised six representatives of national governments—three from low‐ and middle‐income countries, two from large emerging economies and one from a high‐income country—together with two speakers from WHO regional offices, two from WHO headquarters and six technical experts working at national or subnational level. Speaker composition by actor group and broad income context is summarized in the “6. Exploratory” worksheet of Supplementary Data S1. Speakers are identified only by actor group and a sequence number (P01–P16); no personal identifiers are reported.

### 2.2 Analytical Framework

An analytical framework was developed to capture different dimensions of the discourse. Four interrelated dimensions guided the analysis:

1. Challenge framing—how speakers defined and interpreted major challenges facing health systems in the digital era;
2. Principle justification—how speakers justified governance principles and priorities;
3. Transformation pathways—how speakers proposed to achieve health‐system transformation; and
4. Envisioned health systems—how speakers described the desired characteristics of future health systems.

Two additional dimensions were included to capture actor‐specific perspectives: actor type, WHO headquarters, WHO regional offices, national governments, or technical experts; and legitimation strategy, forms of authority used to support policy claims, including empirical evidence, national experience, technical rationality, moral arguments, and institutional authority. The legitimation coding was informed by van Leeuwen's framework, which distinguishes authorization, moral evaluation, rationalization, and mythopoiesis as forms of discursive legitimation [16].

### 2.3 Coding Procedure

The analysis followed a multi‐stage coding process involving open coding, framework validation, full‐text coding, and thematic synthesis. Recurring concepts and themes were first identified inductively from the transcript and subsequently refined through iterative validation across speakers. The validated framework was then applied to the complete dataset to generate a coding matrix linking themes, quotations, actor types, and legitimation strategies. Coding decisions were checked through an independent audit of the full coding matrix and quote bank for consistency, coverage, and alignment between quotations and assigned codes.

### 2.4 Derivation of Governance Principles

Themes were elevated to governance principles when they met three criteria: they were invoked by more than one speaker or actor group; they linked at least two dimensions of the analytical framework, such as challenge framing and transformation pathways; and they expressed a governance orientation rather than a single technical intervention. To connect this derivation to the tiering shown in Table 1, we classified themes as core when they were recurrent, were either cross‐actor or interpretively central, and had a relatively distinct operational pathway. Trust, data protection, and stewardship met the first three criteria for salience, but their operational content was largely embedded in the safety, accountability, and security layers of digital public infrastructure and AI use rather than in a standalone pathway. They were therefore classified as a cross‐cutting supporting condition within P2 and P3, not as a separate sixth principle. This explains why five governance principles were retained while trust and data stewardship were kept visible as an important supporting condition rather than removed from the analysis.

**Table 1:** Major themes and their salience in the WHA79 policy discourse.

Theme	Tier	Speakers (*n* = 16)	Salience	Linked principle(s)
Core				
Equity and access	Core	13	High	P1
People‐centered digital health (augmentation, oversight, data ownership)	Core	12	High	P2
Governance before technology	Core	14	High	P3
Digital public infrastructure and interoperability	Core	12	High	P3, P5
Evidence and context‐sensitivity	Core	11	High (actor‐weighted)	P4
National ownership and sustainable financing	Core	8	Medium (financing actor‐specific)	P5
Supporting				
AI‐assisted services	Supporting	13	High	P2, P4
Service integration and continuity	Supporting	11	High	P1, P3
Trust and data protection	Supporting	9	High (cross‐cutting precondition)	P2, P3
International cooperation and solidarity	Supporting	9	Medium	P5
Supplementary				
Workforce support and capacity	Supplementary	9	Medium	P2
Resilience and security (vision)	Supplementary	9	Medium	P3, P5
Quality and efficiency (vision)	Supplementary	6	Medium	P4
Demographic and epidemiological burden	Supplementary	5	Medium (context)	P1, P4
Digital divide and exclusion	Supplementary	5	Medium	P1
PHC strengthening	Supplementary	5	Medium	P1, P4

*Note:* Speaker counts are unique speakers invoking each theme. Salience integrates prevalence, actor spread, interpretive centrality, and whether a theme had a distinctive operational pathway. Core themes are recurrent and analytically central with relatively distinct pathways; supporting themes are important but operate mainly as cross‐cutting conditions or policy instruments; supplementary themes provide contextual or more actor‐specific evidence.

Abbreviations: P1, equity‐oriented transformation; P2, people‐centered digital health; P3, governance‐led system architecture; P4, evidence‐based and context‐sensitive implementation; P5, national ownership with international cooperation; PHC, primary health care; WHA79, Seventy‐ninth World Health Assembly.

### 2.5 Data Management and Analysis

All coding outputs were documented in a validated database. Representative quotations were preserved verbatim. Quantitative summaries of theme frequency, actor emphasis, and discursive convergence were generated to complement qualitative interpretation. Table 1 reports thematic salience using speaker counts, actor spread, interpretive centrality, and the presence of a distinctive operational pathway. Table 2 links each governance principle to the four‐dimensional analytical framework, including core challenge addressed, justification logic, main pathway, envisioned system attribute, lead actor groups, representative quotation, and discursive convergence. Figure 1 visualizes theme prevalence against discursive convergence, and Figure 2 summarizes actor‐patterned emphasis. These outputs were used as descriptive and interpretive aids, not as inferential statistical tests. Detailed thematic frequency summaries, actor‐by‐theme analyses, and discursive convergence assessments are provided in Supplementary Data S2.

**Table 2:** Master governance principles matrix.

Governance principle	Core challenge addressed (dimension A: challenge framing)	Justification (dimension B: principle justification)	Main pathway (dimension C: transformation pathways)	Envisioned attribute (dimension D: envisioned health systems)	Lead actor groups	Representative quotation	Discursive convergence
P1: Equity‐oriented transformation	Access and geographic inequity; risk that digitalization reproduces exclusion	Equity as the evaluative test of innovation; transformation should reduce, not deepen, disparities	PHC‐first adoption, outreach to underserved groups, service integration and AI screening in low‐resource settings	Equitable and accessible systems that leave no one behind	Ministers/national governments; WHO regional offices; some WHO headquarters framing	“Innovation without access is injustice”	High
P2: People‐centered digital health	Digital exclusion, workforce concerns and the risk that AI substitutes for, rather than supports, people	Digital tools should respond to human needs, protect data rights and augment patients, communities and health workers	AI‐assisted services with human oversight, caregiver/data‐access arrangements and workforce support	People‐centered, empowering and augmentative digital health systems	National governments, WHO regional offices and technical experts; less explicit among WHO headquarters speakers	“AI must support health workers, not replace them”	Moderate
P3: Governance‐led system architecture	Fragmented services, non‐interoperable information systems and disease‐ or tool‐specific digital projects	Governance, standards and institutions should precede and steer technology expansion	Digital public infrastructure, interoperability layers, open standards, identifiers and accountability mechanisms	Integrated, coordinated and resilient health‐system architecture	WHO headquarters and technical experts, supported by several national case examples	“We built tools for diseases, not for systems”	High
P4: Evidence‐based and context‐sensitive implementation	Hype‐reality gap, weak evidence, context mismatch and limited readiness for scale	Innovation should be tested, adapted and scaled only when evidence and local capacity support it	Pilots, evaluation, supervised implementation, external validation and disciplined scale‐up	High‐quality, safe, efficient and context‐sensitive systems	Technical experts and WHO headquarters	“Permissive about experimentation—but disciplined about scale”	Limited/actor‐specific
P5: National ownership with international cooperation	Financing fragility, project‐based fragmentation and dependence on externally supported functions	Transformation should be nationally owned and sustainably financed while supported by international cooperation	Dedicated budget lines, sustainable financing, sovereign digital functions, open standards and collaborative learning	Sovereign, sustainably financed and internationally connected systems	WHO headquarters; LMIC and emerging‐power ministers; regional actors for cooperation and solidarity	“…a sovereign, nationally owned function—as essential as hospital networks…”	Moderate

*Note:* The four content columns correspond to the four dimensions of the analytical framework (challenge framing, principle justification, transformation pathways, and envisioned health systems); each principle draws on all four.

Abbreviations: AI, artificial intelligence; DPI, digital public infrastructure; LMIC, low‐ and middle‐income country; P1, equity‐oriented transformation; P2, people‐centered digital health; P3, governance‐led system architecture; P4, evidence‐based and context‐sensitive implementation; P5, national ownership with international cooperation; PHC, primary health care; WHO, World Health Organization.

**Figure 1:**
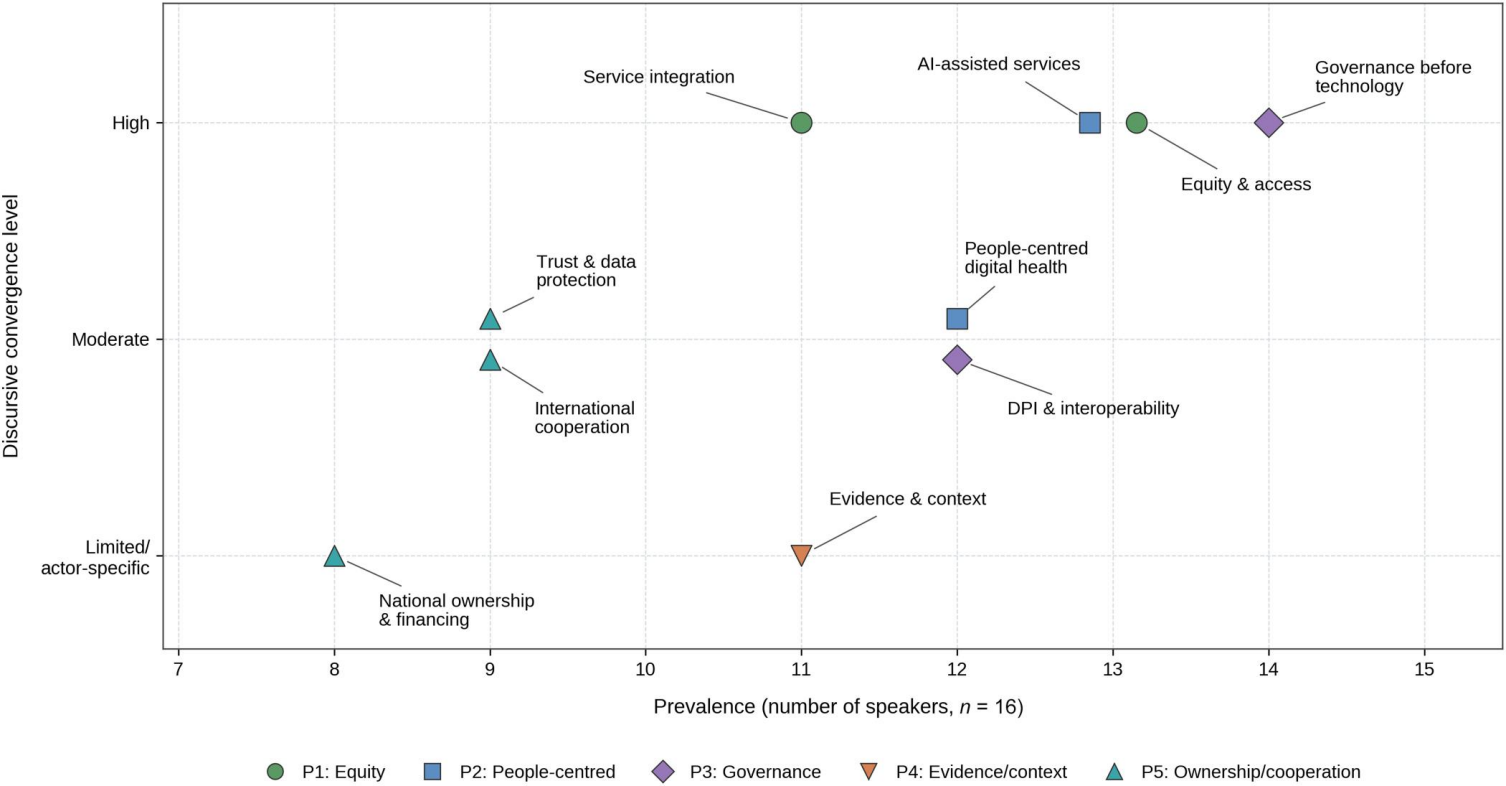
Theme prevalence and discursive convergence in the WHA79 side‐event discourse. Prevalence indicates the number of speakers (*n* = 16) addressing each theme. Discursive convergence was classified descriptively as high, moderate, or limited/actor‐specific. Markers sharing identical coordinates were slightly offset for visibility without changing the underlying values. Colors and shapes distinguish governance principle categories; cross‐cutting themes are shown separately. These classifications do not represent formal policy consensus. AI, artificial intelligence; DPI, digital public infrastructure, P1, equity‐oriented transformation; P2, people‐centered digital health; P3, governance‐led system architecture; P4, evidence‐based and context‐sensitive implementation; P5, national ownership with international cooperation; WHA79, Seventy‐ninth World Health Assembly.

**Figure 2:**
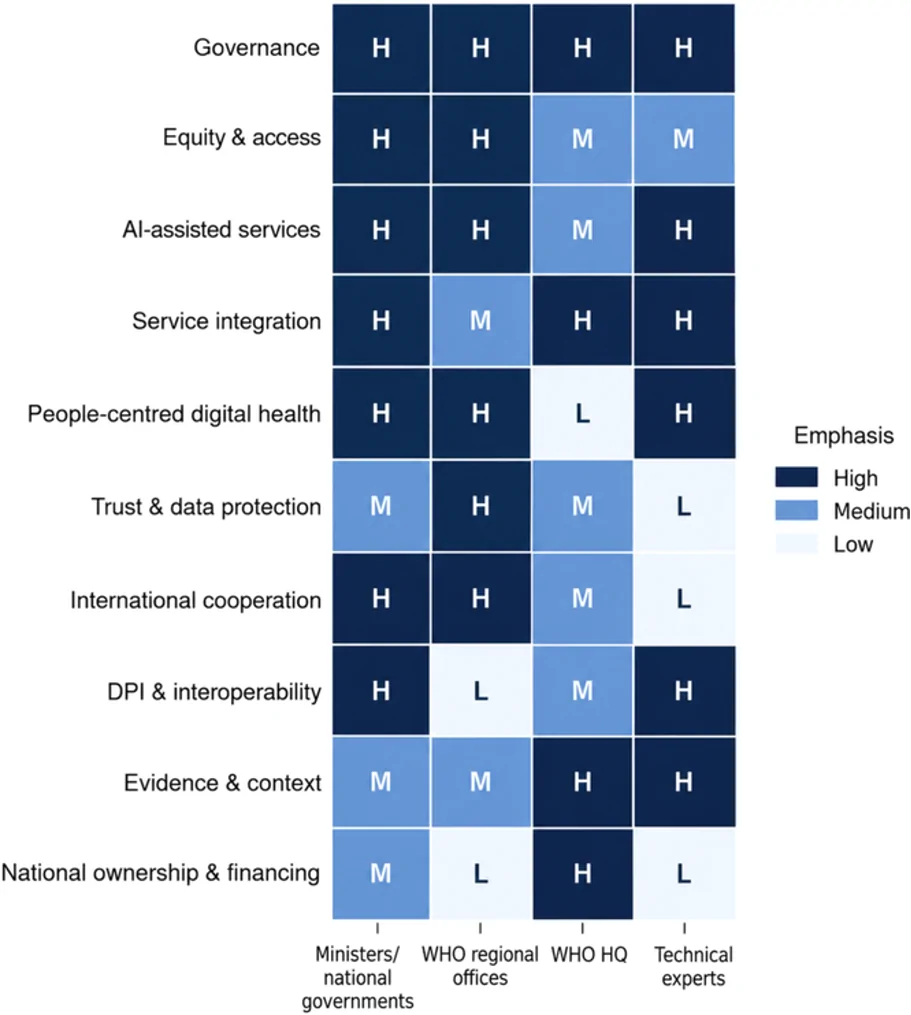
Relative emphasis on governance themes by actor group. Relative emphasis is based on the distribution of coded statements across actor groups and is presented descriptively. AI, artificial intelligence; DPI, digital public infrastructure; H, high; HQ, headquarters; L, low; M, medium; WHO, World Health Organization.

## 3 Results

### 3.1 Challenges to Health‐System Transformation in the Digital Era

The WHA79 official side event discussion highlighted several structural and emerging challenges facing health systems. Equity gaps, service fragmentation, digital exclusion, limited implementation capacity, and pressures from demographic and epidemiological change were recurrently emphasized. Workforce and financing issues also appeared, but they were more actor‐specific: workforce support was a supplementary theme, while financing was concentrated mainly in discussions of national ownership and WHO headquarters discourse. Within the event, speakers broadly converged on the view that health‐system transformation should be understood in the context of these systemic challenges rather than as a purely technological endeavor.

Equity and access emerged as dominant concerns. Participants repeatedly emphasized that reforms should reduce disparities in service provision and utilization across geographic regions, socioeconomic groups, and institutional levels. Technology was positioned as a supportive tool to enhance access, but governance arrangements and policy mechanisms were central to addressing inequities. Fragmentation of services and information systems was another recurring challenge. Disconnected institutional structures, weak coordination, and a lack of interoperability were frequently cited as barriers to effective health‐system performance. These issues were discussed in relation to broader governance and policy frameworks, emphasizing the need for integrated systems.

Speakers also highlighted evolving health demands, including population aging, chronic disease burdens, and service continuity pressures. Effective transformation was described as dependent on governance capacity and strategic planning, with technology functioning as an enabling tool rather than a driver. Concerns regarding implementation capacity, trust, data stewardship, and sustainable financing reinforced the perspective that structural and institutional factors are central to health‐system transformation, while also revealing several areas of more limited or actor‐specific emphasis.

### 3.2 Governance Principles for Health‐System Transformation

In response to the identified challenges, participants articulated five interrelated governance principles that structured the discussion of health‐system transformation. These principles should be read as discourse‐grounded analytical constructs derived from the event, rather than as formal WHO categories or universal recommendations. Technology was consistently framed as a means to support these principles rather than as an independent objective.

Equity‐oriented transformation was highlighted as a foundational principle focused on the distribution of benefits and risks. People‐centered digital health was analytically distinct: it concerned the relationship between digital tools and people, including patients, communities, and health workers, and emphasized augmentation, human oversight, and data rights. Governance‐led system architecture referred to the institutional arrangements, standards, digital public infrastructure, and coordination mechanisms needed for transformation. Evidence‐based and context‐sensitive implementation concerned how reforms should be tested, adapted, and scaled in different settings. National ownership with international cooperation addressed who leads, finances, and sustains transformation, and how international cooperation can support rather than replace domestic leadership.

These distinctions also clarify the boundaries among the principles. Equity‐oriented transformation asks who benefits; people‐centered digital health asks how technology should relate to people; governance‐led system architecture asks what institutional architecture is required; evidence‐based and context‐sensitive implementation asks how reforms should be implemented; and national ownership with international cooperation asks who leads and who supports. Trust, data protection, and stewardship cut across these principles as conditions for people‐centered and governance‐led digital transformation rather than constituting an independent sixth principle, because their operational content was mainly embedded in secure digital public infrastructure, AI oversight, accountability, and data‐governance arrangements.

National ownership combined with international cooperation was presented as essential for sustainable transformation. Domestic leadership and institutional capacity were described as critical foundations, complemented by knowledge sharing, technical assistance, and collaborative learning at the international level.

These principles collectively provided a governance framework linking challenges, justifications, implementation pathways, and envisioned system outcomes. Table 2 summarizes these relationships across the four analytical dimensions and provides representative quotations, while Figure 1 illustrates theme prevalence and discursive convergence within the event.

### 3.3 Pathways Toward Health‐System Transformation

Participants emphasized concrete strategies to operationalize the governance principles. Integrated service delivery, development of interoperable data systems, capacity building, and organizational learning were identified as important pathways. Figure 2 presents the relative emphasis of different actor groups across these themes. Ministers and regional representatives more often foregrounded equity, service delivery, and people‐centered language; WHO headquarters emphasized governance, evidence/context, and financing; and technical experts emphasized responsible AI, digital public infrastructure and interoperability, and evidence‐informed implementation.

A notable pattern was the relative absence of explicit people‐centered language among WHO headquarters speakers, whose discourse more often used system‐architecture, standards, financing, and implementation language. This pattern should not be interpreted as disagreement with people‐centeredness; rather, it suggests that institutional roles shape the register through which shared policy objectives are expressed.

### 3.4 Characteristics of Future Health Systems

The envisioned future health system was defined primarily in governance terms rather than technology terms alone. Participants described systems that are equitable and accessible, people‐centered, integrated across care levels, evidence‐informed, and nationally led while supported through international collaboration. These attributes reflect the operationalization of the governance principles identified earlier and indicate the outcomes participants anticipated from implementing these principles. The detailed mapping of these attributes to challenges, justifications, pathways, actor patterns, and representative quotations is provided in Table 2.

## 4 Discussion

### 4.1 Health‐System Transformation as a Governance Issue

The findings from the official side event indicate that health‐system transformation was primarily conceptualized as a governance challenge rather than a technological endeavor. While digital tools were discussed, participants consistently framed them as instruments to advance broader system objectives such as equity, integration, workforce capacity, and sustainable financing. This perspective aligns with global health governance literature [9, 17, 18, 19], which emphasizes that sustainable improvements depend on institutional arrangements and coordinated policy implementation rather than technology alone. The emphasis on governance highlights the necessity of aligning normative objectives, institutional structures, and implementation mechanisms to achieve effective and equitable health‐system transformation.

Furthermore, the discourse highlighted the interdependence of structural, normative, and operational factors in shaping transformation [10, 11]. For instance, workforce development, data interoperability, and financing mechanisms were linked to governance principles, although with different degrees of salience across actor groups. This pattern illustrates that technological interventions alone are insufficient without accompanying institutional and policy structures [12, 13, 14]. It reinforces the argument that health‐system transformation is fundamentally a governance project, where digital tools serve as enabling instruments rather than the primary drivers of change. These interpretations are intended to make sense of the discourse patterns observed in this event, rather than to infer individual speakers' motivations or to establish how such discourse may influence policy implementation in practice.

### 4.2 Emergence and Justification of Governance Principles

From a discursive institutionalist perspective, governance principles are constructed and legitimized through policy dialog rather than existing as pre‐existing entities [8, 20]. The official side event served as a forum where actors collectively defined challenges, justified priorities, and articulated desired futures for health systems. The five governance principles emerged through this process as an integrated discursive framework rather than as isolated topics. The analysis therefore contributes less by proposing wholly new normative principles than by showing how established health‐system, digital health, and AI‐governance norms were recombined and operationalized in a multilateral setting.

The comparison with existing frameworks, summarized in the “5. Framework Alignment” worksheet of Supplementary Data S1, indicates that most of the identified principles are not entirely new. Rather, they largely restate or extend established commitments in health‐system strengthening, digital health, and AI governance. The most distinctive emphasis is P5: digital capability was framed not simply as a technical resource or implementation tool, but as a sovereign public function requiring domestic stewardship, dedicated financing, and international cooperation [11, 12, 13, 14, 21].

This framing may reflect the increasingly infrastructural role of digital capability in health systems. As digital identifiers, interoperability layers, data‐governance arrangements, and AI‐enabled services become embedded in service delivery, surveillance, financing, and quality improvement, they become harder to manage as isolated projects. They instead require public oversight, sustained budgetary commitment, and institutional accountability.

The analysis also points to several areas that were relatively muted in the side event discourse despite their prominence in theoretical and normative literature. These include enforcement and accountability mechanisms, private‐sector and platform power, procurement and vendor‐dependence risks, and legal redress when digital or AI‐enabled systems fail. This absence is informative. The event discourse was stronger in articulating positive principles for alignment than in addressing conflict, noncompliance, and power asymmetries. Official side events may therefore be useful venues for norm articulation and policy convergence, while being less likely to surface the contested politics of implementation.

The findings suggest that the principles both reflected shared norms and were actively shaped by multi‐actor interactions. Speakers used empirical evidence, national experiences, moral reasoning, technical rationales, and institutional authority to legitimize proposed actions [16, 22]. Several tensions were also visible. Governance operated as a shared signifier, yet actors attached different operational meanings to it; WHO headquarters tended to use more technocratic architecture and financing language, whereas national and regional actors more often foregrounded equity and people‐centered service delivery. Evidence was invoked both as a reason to scale innovation and as a reason to restrain premature scaling. These tensions constitute a more specific contribution of the dataset beyond the general observation that governance matters.

### 4.3 Discursive Convergence in Multilateral Policy Discourse

The analysis illustrates that official side events can function as institutionalized platforms for norm diffusion, agenda‐setting, and alignment among governments, WHO representatives, and technical experts. While actor‐specific emphasis varied, Figures 1 and 2 demonstrate substantial discursive convergence around the five governance principles within this event. These findings are consistent with literature on orchestration, epistemic communities, and political priority formation in global health [23, 24, 25]. At the same time, discursive convergence should be interpreted cautiously. In a diplomatic setting, speakers may emphasize shared normative language because of situational expectations, institutional roles, or the consensual style of multilateral forums. The observed convergence therefore indicates alignment in this event‐specific discourse, not necessarily universal theoretical acceptance, formal policy agreement, or implementation commitment.

### 4.4 Implications for Health‐System Governance and Policy Practice

The study has several implications for policy and practice. First, policymakers should regard governance principles as central levers when implementing health‐system transformation, ensuring that technological interventions are embedded within coherent institutional frameworks. Second, WHO and other multilateral organizations can use official side events to facilitate norm dissemination, knowledge exchange, and alignment among member states, while avoiding the assumption that rhetorical alignment alone guarantees implementation. Third, the principles have different implications across contexts. For low‐ and middle‐income countries, they highlight opportunities to use digital tools to extend access, while underscoring infrastructure, workforce, financing, and dependence risks. For high‐income countries, they point to the need to manage digital exclusion, privacy, safety, and over‐automation. For emerging powers, they suggest opportunities for regional cooperation, digital public infrastructure development, and South‐South learning, alongside challenges related to interoperability, sovereignty, and trust. For WHO, they reinforce the value of standard‐setting and convening functions, while also highlighting the need to support country ownership and sustainable financing.

Additionally, the findings suggest that actor heterogeneity may shape policy discourse. The different emphases of governments, WHO representatives, and technical experts reflect varying institutional roles, policy responsibilities, resource positions, and contextual priorities. This heterogeneity highlights the need for multilateral governance processes to accommodate plurality while seeking shared understanding.

### 4.5 Limitations

Several limitations should be noted. First, the analysis is based on a single official side event and should therefore be interpreted as an event‐specific account rather than as representative of all WHA deliberations or wider global policy discourse. Second, although the coding process was audited for consistency and coverage, the interpretation of policy discourse inevitably involves researcher judgment. Third, because the study did not include interviews with speakers or follow‐up analysis of policy implementation, it cannot determine how speakers themselves understood particular statements, or whether the principles identified in the discourse subsequently influenced policy design or institutional practice. Fourth, anonymizing actors helped reduce attribution risk, but it also limited the extent to which individual statements could be linked to specific institutional decision‐making processes. Finally, the study examines discourse as a window into governance principles, rather than as evidence of implementation. Future research could compare multiple WHA events, formal resolutions, national policy documents, and implementation cases to assess how such principles are translated into policy design, financing arrangements, and practice.

## 5 Conclusions

This study highlights how governance principles for health‐system transformation are articulated, justified, and operationalized in multilateral policy discourse. Official side events provide an important forum for negotiating and aligning priorities among governments, WHO, and technical experts. The findings underscore the central role of governance frameworks in enabling equitable, integrated, and sustainable health systems, while showing that digital technologies operate mainly as enabling instruments.

## Author Contributions

**Hui Yin:** conceptualization, methodology, formal analysis, data curation, writing – original draft. **Xiaobaiju Wu:** data curation, validation, writing – review and editing. **Daping Song:** data curation, validation, writing – review and editing. **Yunping Wang:** data curation, validation, writing – review and editing, supervision. **Xiaoyun Liu:** methodology, validation, writing – review and editing. **He Zhu:** methodology, validation, writing – review and editing. **Xueqing Tian:** data curation, validation, writing – review and editing. **Xiaodan Fan:** data curation, validation, writing – review and editing. **Yingpeng Qiu:** data curation, validation, writing – review and editing. **Minghui Ren:** conceptualization, methodology, writing – review and editing, supervision, funding acquisition.

## Ethics Statement

Ethics approval was not sought because this study analyzed an official policy‐event transcript and did not involve patients, personal health information, or human‐subject intervention. Speakers were analyzed by actor group rather than by personal identity, where possible to reduce attribution risk.

## Consent

Informed consent was not required because the study was based exclusively on the transcript of an official World Health Assembly side event and involved no direct interaction with participants or collection of private or personal health information.

## Conflicts of Interest

The authors declare no conflicts of interest.

## Supporting information


**Supplementary Data S1**: Coding framework and governance principles mapping. Contains the codebook, master coding matrix, quotation bank, thematic frequency summaries, governance principles mapping, comparison with existing normative frameworks, and exploratory actor‐group analysis.

**Supplementary Data S2**: Thematic and actor‐patterned discourse analysis. Contains the coding key, quotation bank, theme frequency summaries, actor‐by‐theme matrix, and discursive convergence and divergence analysis.

## Data Availability

The coded analytical matrices and the representative quotation database are provided in the supplementary materials. The full transcript is not publicly deposited because it contains identifiable statements from a policy event.
